# Females are the brighter sex: Differences in external fluorescence across sexes and life stages of a crab spider

**DOI:** 10.1371/journal.pone.0175667

**Published:** 2017-05-03

**Authors:** Erin E. Brandt, Susan E. Masta

**Affiliations:** 1 Department of Environmental Science, Policy and Management, University of California, Berkeley, California, United States of America; 2 Department of Biology, Portland State University, Portland, Oregon, United States of America; University of Vienna, AUSTRIA

## Abstract

Fluorescence is increasingly recognized to be widespread in nature. In particular, some arachnids fluoresce externally, and in spiders the hemolymph fluoresces. In this study, we examined the external fluorescence and the fluorophores of different sexes and life stages of the crab spider *Misumena vatia* (Clerk 1757), a sit-and-wait predator that feeds on insects as they visit flowers. We designed novel instrumentation to measure external fluorescence in whole specimens. We found that although males and females possess internal fluorophores with similar properties, the external expression of fluorescence varies across sexes and life stages. Spiders fluoresce brightly as immatures. Females maintain their brightness to adulthood, whereas males become increasingly dim as they mature. We suggest that external fluorescence likely contributes to visual signaling in these animals, and that it differs between the sexes as a result of differences in foraging ecology and behavior.

## Introduction

Fluorescence is a phenomenon characterized by the emission of light by a substance following excitation by a shorter wavelength of light. Fluorescence occurs when electrons of molecules known as fluorophores become excited to a higher electronic state upon encountering short wavelengths, and subsequently fall back to the ground state. Many natural substances (e.g., certain minerals) fluoresce, and there are examples in biological systems as well (reviewed in [1]). Fluorophores have been found in a diversity of marine organisms, including corals [2], crustaceans such as mantis shrimp [3], and fish [4, 5]. Fluorophores appear to be much less common among terrestrial taxa, although at least one species of parrot [6], some insects [7], and some arachnids [8–10] have been found to fluoresce.

Although fluorophores may be present within an animal’s tissues or circulatory system, these fluorophores will not fluoresce unless exposed to excitation wavelengths. Andrews et al. [8] showed that all spiders they tested possessed fluorophores in their hemolymph, but that only some produced bright external fluorescence. The expression of bright external fluorescence appears to have evolved multiple times among spider lineages, as the distribution of fluorescing taxa is spread across a phylogenetic tree of spider relatedness [8]. However, the functional significance of this externally expressed fluorescence is unknown for the majority of species.

In one species of jumping spider, *Cosmophasis umbratica*, it has been shown that externally expressed fluorescence is involved with sexual signaling [11]. Because *C*. *umbratica* is a visually acute jumping spider, sexual selection via visual courtship displays likely shaped the expression of fluorescence in this species. It is possible that other species of spiders may use fluorescence in sexual signaling, but most spiders have only fair to poor vision and do not use visually mediated courtship behavior [12, 13]. For instance, although the crab spider *Misumena vatia* (Clerck 1757) can likely perceive colors, it lacks the visual acuity of jumping spiders [14, 15]. Therefore, it seems unlikely that fluorescence would be involved in mate choice for taxa, like *M*. *vatia*, that are less visually acute. Instead, in spider species exposed to the sun (and thus to excitation wavelengths for fluorophores), externally expressed fluorescence could potentially serve other functions, such as camouflage or prey attraction.

To help clarify the nature of expressed fluorescence in spiders and to begin to assess its potential function(s), we aimed in this study to examine fluorescence in the crab spider *M*. *vatia* in the context of sex, life stage, and foraging ecology. We chose *M*. *vatia* because this species fluoresces brightly [8] and also varies considerably in size and foraging ecology across sexes and life stages. This variation has been well-studied and documented [16]. At the same time, most existing research relating to optical qualities of spiders and their prey, predators, and substrates has dealt only with adult females, so we aimed with our approach to gain insights by providing wider context.

*Misumena vatia* is a diurnal sit-and-wait predator and is often found on flowers where it preys upon pollinating insects [17, 18]. Like the adult females of many spiders, those of *M*. *vatia* invest in capturing large prey items, which provide energy allowing them to produce a large clutch of eggs [19]. Immature spiders have similar feeding habits, but typically cannot subdue large prey, and instead feed on small insects [20]. Adult males eat little and spend a large proportion of their time searching for females [16, 21]. The diurnal habits of *M*. *vatia* cause it to be frequently exposed to fluorophore-exciting wavelengths generated by the sun.

In this study, we aimed to determine whether fluorescence changes over the course of a spider’s life, and whether fluorescence differs between males and females. We hypothesize that significant variation between sexes or among age classes could indicate that selection favors differential expression of fluorescence according to developmental stage or according to sex.

To obtain detailed data on externally expressed fluorescence in *M*. *vatia*, we developed equipment specifically tuned to capture emission wavelengths from one specific fluorophore we found in *M*. *vatia*. We find that fluorescence expression changes over the course of a male spider’s lifetime, becoming less expressed as the spider reaches sexual maturity. We present these data and seek to frame the information in an ecological context by identifying several hypotheses regarding the nature and scale of fluorescence across sexes and life stages in *M*. *vatia*.

## Materials and methods

### Specimen collection

*Misumena vatia* specimens were collected during the spring, summer, and early fall of 2007–2011 from Oregon and southern Washington, USA. All spiders were collected from public forestlands, or in public rights-of-way along roadsides, where permits were not required to collect spiders. These spiders are common, and are not protected by law. *Misumena vatia* individuals are able to reversibly change color from white to yellow, but for this study we utilized only white individuals, as we were interested in differences in fluorescence across sexes and life stages, rather than differences in fluorescence related to color morph. Spiders were maintained in the lab in clear plastic 7-dram vials (from United States Plastic Corp.) on a 12:12 light cycle at room temperature and fed fruit flies or crickets twice per week, until they reached the appropriate stage of their life cycle for our analyses. Spiders were euthanized by freezing at -80°C in microcentrifuge tubes and maintained at that temperature for at least one full day prior to imaging.

### Fluorophore extraction

We characterized the spectral characteristics of fluorophores found in *M*. *vatia*, following the protocol in Andrews et al. [8]. In brief, we collected fluorophores from the abdomens of adult males (n = 3) and females (n = 5) that were previously assayed for external fluorescence intensity. We ground entire abdomens in 95% ultrapure ethanol and allowed each sample to sit for 48 hours in the dark to extract fluorophores. Each sample was then centrifuged to pelletize any solid material and the resulting supernatant was used for fluorometry analysis. All fluorometry was performed in the laboratory of Dr. Scott Reed at the University of Colorado Denver using a PTI Spectrofluorometer.

### Fluorescence light source determination and calibration

Fluorophores typically have discrete peaks of excitation and emission wavelengths that are unique to each fluorophore. We used the peak excitation data from the *M*. *vatia* fluorophore extractions to guide our selection of appropriate wavelengths of light necessary to excite the spiders’ externally expressed fluorophores. The generation of precise excitation wavelengths was thus an important aspect of eliciting biologically significant levels of external fluorescence. Light-emitting diodes (LEDs) produce a fairly narrow band of wavelengths, unlike broad-spectrum light sources such as xenon-arc lamps. We therefore used singly a series of ultraviolet-emitting LEDs as our light sources to excite fluorescence in spiders. This use of narrow ranges of wavelengths for excitation eliminated the need for filters that block undesired wavelengths.

We determined that peak excitation of *Misumena* fluorophores occurs at excitation wavelengths of approximately 290 nm and 330 nm. For this study, we focused specifically on quantifying externally expressed emission from excitation that occurs at 330 nm. This wavelength is present in the light from the sun that would reach a diurnal spider, so emission measured at this wavelength would be seen in nature [22]. LEDs were available only at some specific wavelengths, and we experimented with several different LEDs to determine which one elicited maximal fluorescence. For the purposes of this paper, we will focus on data from images captured with the 340-nm LED (LED340W, Thorlabs, Newton, NJ, USA) as fluorophores exhibited maximum excitation closest to this wavelength.

To calculate exposure times for the fluorescence images, we measured the spectral curve (absolute irradiance) of both the LED (S1A Fig) and of a typical light regime (S1B Fig) in a representative spider habitat. Comparing the two, we were able to calculate how many seconds of LED exposure would expose the spider to the same number of photons as 0.1 second of sun exposure at 340 nm. We calculated an exposure time of 117 seconds.

### Instrumentation design

Quantifying fluorescence in a whole specimen (as opposed to a fluorophore extract) can be technically challenging. Color (reflectance) is typically measured using spectrophotometry. However, there is no way to tell the difference between reflected wavelengths and those emitted by fluorophores in a typical spectrometry setup. It is possible to mask a specimen at the excitation wavelengths so that fluorescence is suppressed and only reflectance is observed [23]. However, it is then impossible to determine the level of reflectance at those excitation wavelengths due to non-fluorophore pigments. In *M*. *vatia*, the excitation wavelengths are in the UV. Because UV reflectance is important to the visual systems of *M*. *vatia*’s prey [24], masking those wavelengths to stop fluorescence would also remove a vital portion of the reflectance spectrum.

To work around this issue, we used a dual approach with both spectrometer measurements and carefully filtered photographs to assess overall coloration and fluorescence, respectively. A challenge with using such a method is that the units of the two types of measurement are not directly comparable. However, this technique enabled us to precisely isolate light due strictly to fluorescence so that we could make specific hypotheses about only fluorescence. In the case of *M*. *vatia* we compared the relative reflectance of different sexes and life stages at the known emission peaks of the fluorophores with relative fluorescence intensity from photographs.

An additional challenge in designing instrumentation for measuring spider fluorescence is that spider fluorophores are excited in the UV range of light, but emit wavelengths in both the UV and visible portions of the spectrum [8]. Ultraviolet light does not pass through most standard optics without some degree of attenuation and auto-fluorescence. This makes fluorescence emanating from the study subject difficult to discern and quantify [2]. Therefore we were limited to measuring fluorescence emission in the visible range.

We captured images with a model MVX10 stereo macroscope transparent to near UV with a 1x objective (Olympus America Inc, Center Valley, PA, USA). The microscope was connected to a UV-sensitive Orca R2 camera (Hamamatsu Corporation, Bridgewater, NJ, USA), which produced 12-bit black-and-white images. The microscope was outfitted with a custom-built filter adapter to direct light to and from the specimen (S2 Fig). As a light source, we used a 340-nm LED connected to a variable DC power supply.

We affixed an optical dichroic beam splitter filter with a transmission band of 420–750 nm inside the custom-built filter holder apparatus. The beam splitter provided a filtering step by directing excitation wavelengths to the specimen and allowing only the resulting emission wavelengths to pass through to the camera. Our instrumentation also included an optional second blocking filter above the dichroic beam splitter filter, which blocked wavelengths less than 450 nm from reaching the camera. We initially used this filter, before deciding on using only the dichroic filter. The filters ensured that resulting images were representative only of fluorescence, rather than of stray excitation wavelengths or reflectance. One advantage of our wavelength filtering apparatus is that light sources and filters can be easily swapped for different wavelengths if desired. Additionally, because we had complete knowledge of which wavelengths were able to pass through to the camera, we had no need for color images and were able instead to use an ultra-sensitive black-and-white camera.

### Fluorescence photography

For measurements of fluorescence from spiders, 15 adult females, 9 adult males, 4 penultimate females, 9 penultimate males, and 9 immature spiders were photographed with the optional blocking filter in place. A subset of adults (10 adult females, 4 adult males) was photographed with only the dichroic filter, allowing wavelengths of 420 nm and above to reach the camera. We allowed specimens to defrost for several minutes after removing them from the -80°C freezer. Spiders were then pinned into a standardized position to eliminate variance in exposure to the light source. We pinned spiders into place (without piercing the specimen, so as to prevent fluorescent abdominal contents from leaking out) to a substrate consisting of closed cell foam covered by clean, lint-free black velvet.

All subsequent measurements were made in a darkroom. We took spectrometer readings immediately after the animal was pinned into position. Next, the specimen was placed under the microscope, brought into focus, and photographed under white light. All images were taken at 1.6x magnification of the objective, for a total magnification of 16x. After a white-light image was taken, the UV light source was powered on and a fluorescence image was captured.

After all photographs had been captured, we repositioned the spider to bring another body part into focus, until all body parts were photographed. We photographed the dorsal aspect of the abdomen, cephalothorax, and the first two pairs of legs. Legs were separated from the animal before being photographed, and the femur was always used as the region for maximal focus of the image. As previously stated, hemolymph or associated body liquids fluoresce brightly, so we did not image any body part that had suffered damage during processing. Following completion of all imaging, each specimen was stored in the—80°C freezer in 95% ultrapure ethanol for later fluorophore extraction.

### Image analysis

We conducted all image analysis using the ImagePro 7 Plus^®^ software package (Media Cybernetics, Inc, Rockville, MD, USA). To determine the brightness of the fluorescence we used a measure termed “pixel intensity”, based on the encoding of black-and-white images, to determine the brightness of each pixel. Black-and-white 12-bit images encode 4096 levels of gray. Therefore, a pixel with a numerical value of 0 indicates that the pixel is completely black and a pixel with the value of 4095 is completely white.

To analyze a series of images, we first loaded the white light image into ImagePro^®^. Next, an “area of interest” (AOI) was traced around the areas of the image to be analyzed. This tracing process was performed using the white light image because if a given specimen did not fluoresce brightly, it was difficult to see and trace the necessary areas of the image in fluorescence photographs.

We drew three AOIs for each body part imaged. When all AOIs had been drawn and saved, all fluorescence images were loaded into ImagePro^®^, and a macro was run that automatically applied each AOI to each image in sequence and performed measurements and calculations based on the circled region of the photograph. These calculations were exported to a.txt file to be processed later.

We used two main types of measurements to analyze all fluorescence image data: pixel intensity and percent in brightest category. First, the average pixel intensity (brightness) for each body region was calculated for each individual. Then, to calculate percent in brightest category, we divided the pixel intensity range into ten categories of brightness, with each category representing an equal tenth of the possible spread of pixels. These ten categories were then presented as a histogram. We divided these ten classes of pixel intensity further into three categories: dim, medium, and bright. This closely matched the process by which fluorescence intensity was quantified in images previously taken by Andrews et al. [8]. Then, we calculated the percentage of area in a given body part that was occupied by pixels in the brightest category. The optional blocking filter blocked more light from reaching the camera than using the dichroic beam splitter filter alone, and therefore made quantifying the percent of each body part that fluoresced the brightest more difficult, hence we focused our analyses of this using only data gathered with the beam splitter filter alone.

All statistics were performed using R. Due to small sample sizes and zero-inflated datasets (see Results for further explanation), we used non-parametric tests to analyse the data. To compare brightness in average pixel intensity between sexes and life stages, for each of three body parts, Kruskal-Wallis tests were performed, followed by post-hoc Dunn’s tests, where appropriate. These analyses were performed for data gathered with and without the optional blocking filter. We used the same statistical methods to analyze the data for percent in brightest category that were gathered without the blocking filter.

### Reflectance measurements

We also took reflectance measurements of spiders that were analyzed for external fluorescence (n = 15 adult females, 5 adult males, 3 penultimate females, 9 penultimate males, and 2 immatures). This was done to help determine what role fluorescence plays in the overall visual signal displayed by the spiders. Reflectance measurements were taken using a USB4000 spectrometer with a DH-2000 light source (Ocean Optics, Dunedin FL, USA) connected to a Dell laptop computer running Windows XP and Ocean Optics’ SpectraSuite^™^ software. We used an integration time of 0.007 s, and held the probe 2 mm from the specimen, at an angle of 90° relative to the frontal plane of the spider. Ten readings were averaged together per measurement. We repeated measurements four times for each specimen.

Whenever possible, reflectance measurements were taken from each spider that was photographed and assessed for fluorescence. However, the small size of some adult males and all immature spiders decreased the accuracy of some of these measurements.

The resulting reflectance data could be assessed for significant differences with further data transformation [25]. However, our goal was to use the reflectance data to provide a general comparison between overall visual signal and fluorescence. Thus, reflectance data are presented in a qualitative manner.

## Results

### Average fluorescence intensity

**Fig 1:**
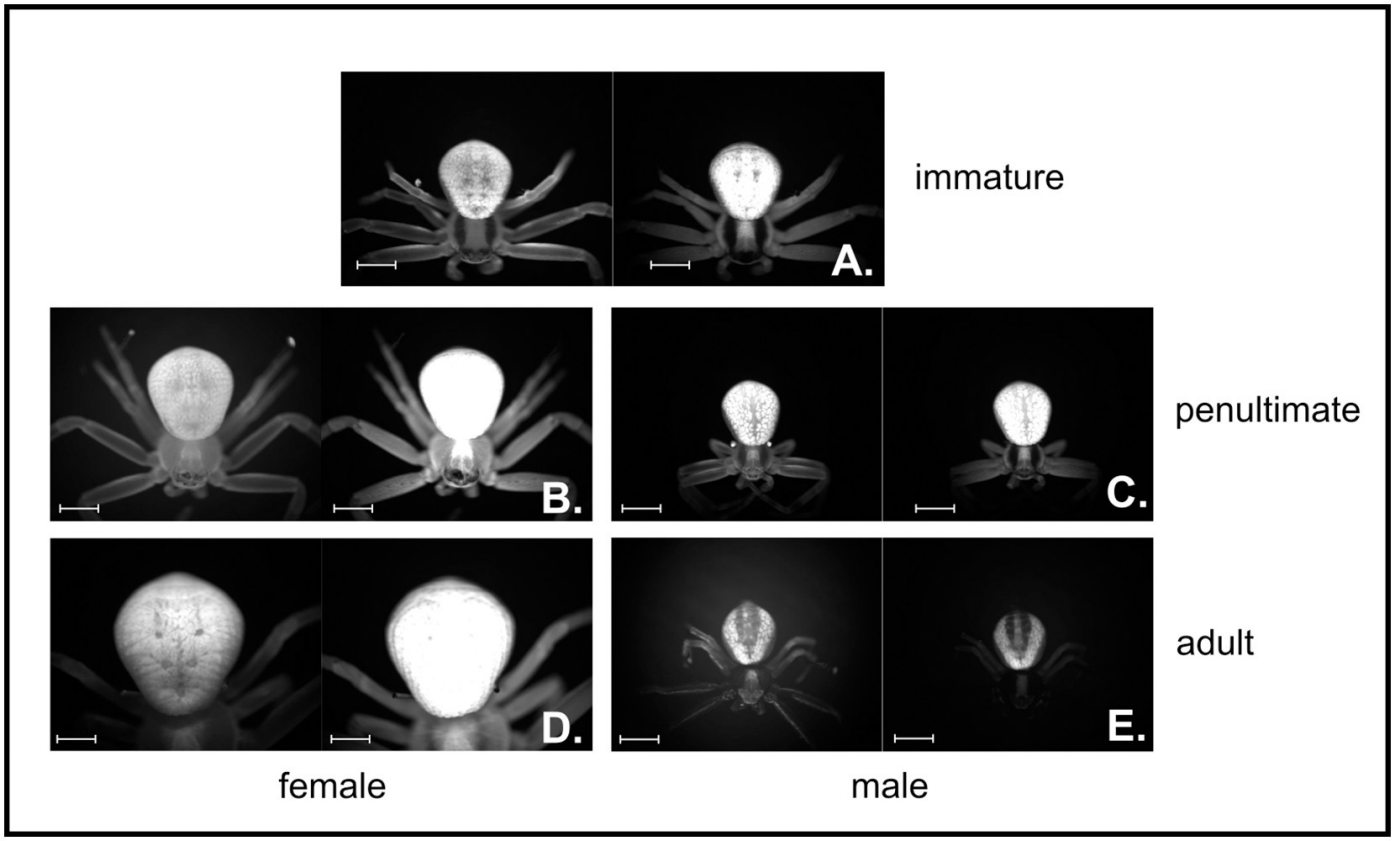
Representative images of *M*. *vatia* abdomens under white light and 340-nm light. Each panel shows the same individual photographed under white light (left) and 340-nm light (right). Images are shown for (A) immature, (B) penultimate female, (C) penultimate male, (D) adult female, and (E) adult male specimens. Bar length = 1 mm.

**Fig 2:**
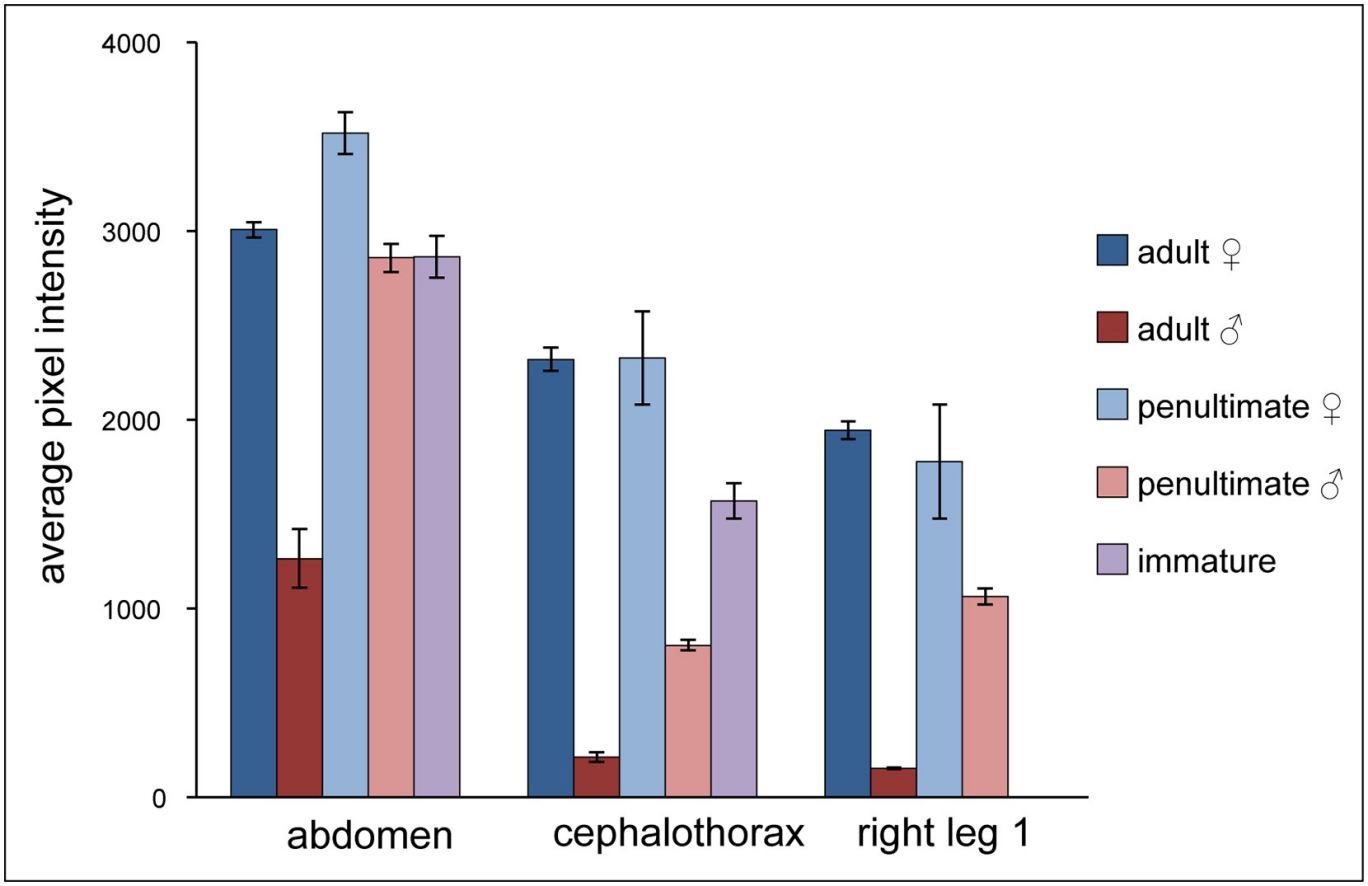
Average fluorescence intensity for abdomen, cephalothorax, and right leg 1 of *M*. *vatia*. Within a body part, significant differences in fluorescence intensity are shown in Tables 1 and 2. Right leg 1 was not included for immature spiders due to their small size.

Spiders of all age classes emitted wavelengths of light in the range visible to humans when illuminated with a 340-nm light (Fig 1). As a general trend, adult males were substantially dimmer than females (Fig 2). Fluorescence intensity differed significantly among sexes and among life stages, both in images with and without the use of the blocking filter (Fig 2; see Table 1 for Kruskal-Wallis results, Table 2 for p-values associated with post-hoc Dunn’s tests, and S1 and S2 Tables for results using the blocking filter. The data used to generate these figures and tables is provided in S1 Dataset). We focus discussion of our results on images obtained using only the beam splitter filter, for which we had smaller sample sizes and fewer significant differences in fluorescent intensity. However, the beam splitter filter alone allowed a greater amount of fluorescence to reach the camera, and therefore more readily allowed categorization of pixel intensity.

**Table 1:** Kruskal-Wallis results for average fluorescent intensities and percent in brightest category of all body parts, using only the beam splitter filter. * indicates p < 0.05. (Adult females n = 10; adult males n = 5; penultimate females n = 4; penultimate males n = 9; immature spiders n = 9).

**Average Intensity**	X^2^	X^2^_crit_	df	p
abdomen	12.08	9.48	4	0.017*
cephalothorax	24.88	9.48	4	<< 0.001*
right leg 1	15.23	7.81	3	0.004*
**Percent in Brightest Category**	X^2^	X^2^_crit_	df	p
abdomen	10.72	9.48	4	0.029*
cephalothorax	19.72	9.48	4	<< 0.001*
right leg 1	5.91	7.81	3	0.120

**Table 2:** Results of post-hoc Dunn’s tests for differences in fluorescence intensity of each body part. Legs of immature spiders were not imaged due to small size. * indicate p < 0.05.

**abdomen**	adult male	penultimate female	penultimate male	immature
adult female	0.063	1.000	0.989	1.000
adult male	-	0.009*	0.108	0.063
penultimate female		-	0.822	1.000
penultimate male			-	1.000
**cephalothorax**	adult male	penultimate female	penultimate male	immature
adult female	<< 0.001*	0.754	0.003*	0.318
adult male	-	0.011*	0.591	0.058
penultimate female		-	0.083	0.591
penultimate male			-	0.340
**right leg 1**	adult male	penultimate female	penultimate male	
adult female	0.002*	1.000	0.274	
adult male	-	0.034*	0.192	
penultimate female		-	0.934	

In comparisons for each of three body regions, the cephalothoraxes and legs of adult female *M*. *vatia* individuals fluoresced significantly more brightly than those of adult males when excited with a 340-nm light (Fig 2 and Table 2). The abdomens of adult females were also brighter than those of adult males, but differed significantly only at the p = 0.10 level. Penultimate-stage female spiders were consistently brighter in their fluorescence than adult male spiders, showing significant differences for each of the three body regions (Fig 2 and Table 2).

Data from the abdomens of spiders showed fluorescence intensity of penultimate male and immature spiders to be similar to that of adult female spiders (Fig 2). For spiders’ cephalothoraxes, adult females and penultimate females displayed similar levels of fluorescence, and both were significantly brighter than adult male spiders (Fig 2 and Table 2). Additionally, the cephalothoraxes of adult females fluoresced significantly more brightly than those of penultimate males. The legs of adult and penultimate female spiders were each significantly brighter than adult males (Fig 2 and Table 2)

### Differences in fluorescence within body regions

**Fig 3:**
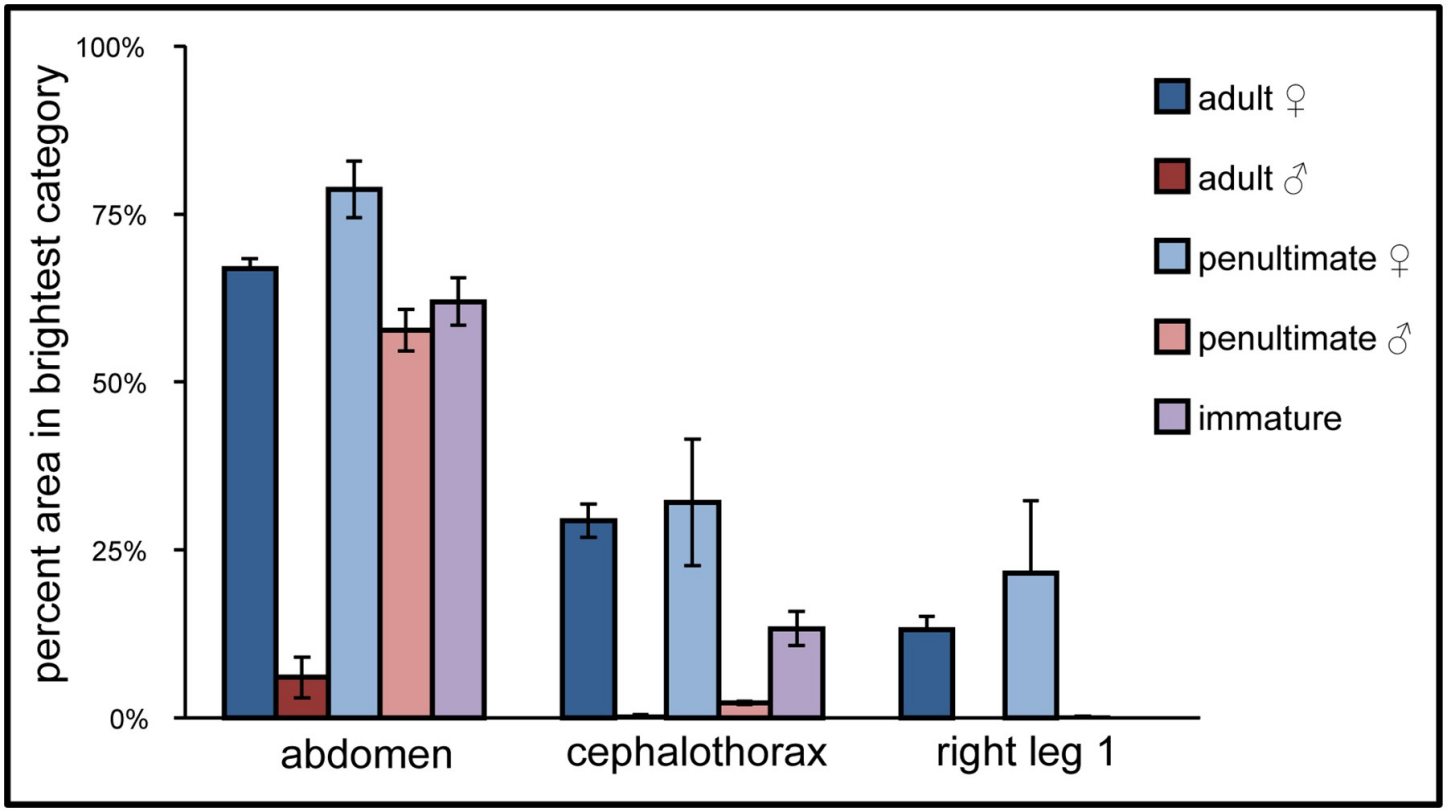
Percent area in brightest category in abdomen, cephalothorax, and right leg 1 of *M*. *vatia*. (Adult females n = 10; adult males n = 4; penultimate females n = 4; penultimate males n = 9; immatures n = 9). Right leg 1 was not included for immature spiders due to their small size.

Because a spider’s brightest fluorescing areas likely contribute the most to its overall perceived coloration, we determined, for each body region, the percent of surface area that fluoresced especially brightly. Spiders varied in the extent of body surface that fluoresced in our brightest measurement category, as measured by pixel intensity. As with the data on average pixel intensity described above, for each body region, adult females showed a consistently greater percent area in the brightest category than adult males, and penultimate females showed consistently more percent area in the brightest category than penultimate males (Fig 3), although some differences did not attain statistical significance (see Table 3). Penultimate females also showed greater percentages of body surface in the brightest pixel intensity category than did adult males (Fig 3).

**Table 3:** Results of post-hoc Dunn’s tests for percent of body part in brightest category. Only abdomen and cephalothorax data are shown because there were no significant differences among legs. * indicates p < 0.05.

**abdomen**	adult male	penultimate female	penultimate male	immature
adult female	0.119	1.000	1.000	1.000
adult male	-	0.020*	0.156	0.066
penultimate female		-	1.000	1.000
penultimate male			-	1.000
**cephalothorax**	adult male	penultimate female	penultimate male	immature
adult female	0.006*	0.938	0.007*	0.280
adult male	-	0.040*	0.846	0.321
penultimate female		-	0.080	0.594
penultimate male			-	0.594

For the abdomen, no statistical differences were seen, except between penultimate females and adult males (Table 3). For the cephalothorax adult males had significantly less area that fluoresced brightly, as compared with both penultimate and adult females. For the legs, levels of fluorescence did not differ significantly, in part due to small sample size of adult males measured, but mean values can be seen in Fig 3. The fluorescence of the legs of immature spiders was not measured, as the legs were often too small to remove without damaging them, and our instrument did not allow quantification of areas of such small size.

Although differences in the brightness of fluorescence, and in the surface area that fluoresced the most brightly, seem clear and substantial in graphical form (Figs 2 and 3), many of these differences did not attain statistical significance. Beyond modest sample sizes, one reason for this is that some data were zero-inflated, with a large number of zero values. For example, many males had no body regions in the brightest category, and hence received a value of zero. Zero-inflation skews the data away from a normal distribution, requiring non-parametric statistical tests that can be conservative in detecting significant differences.

**Fig 4:**
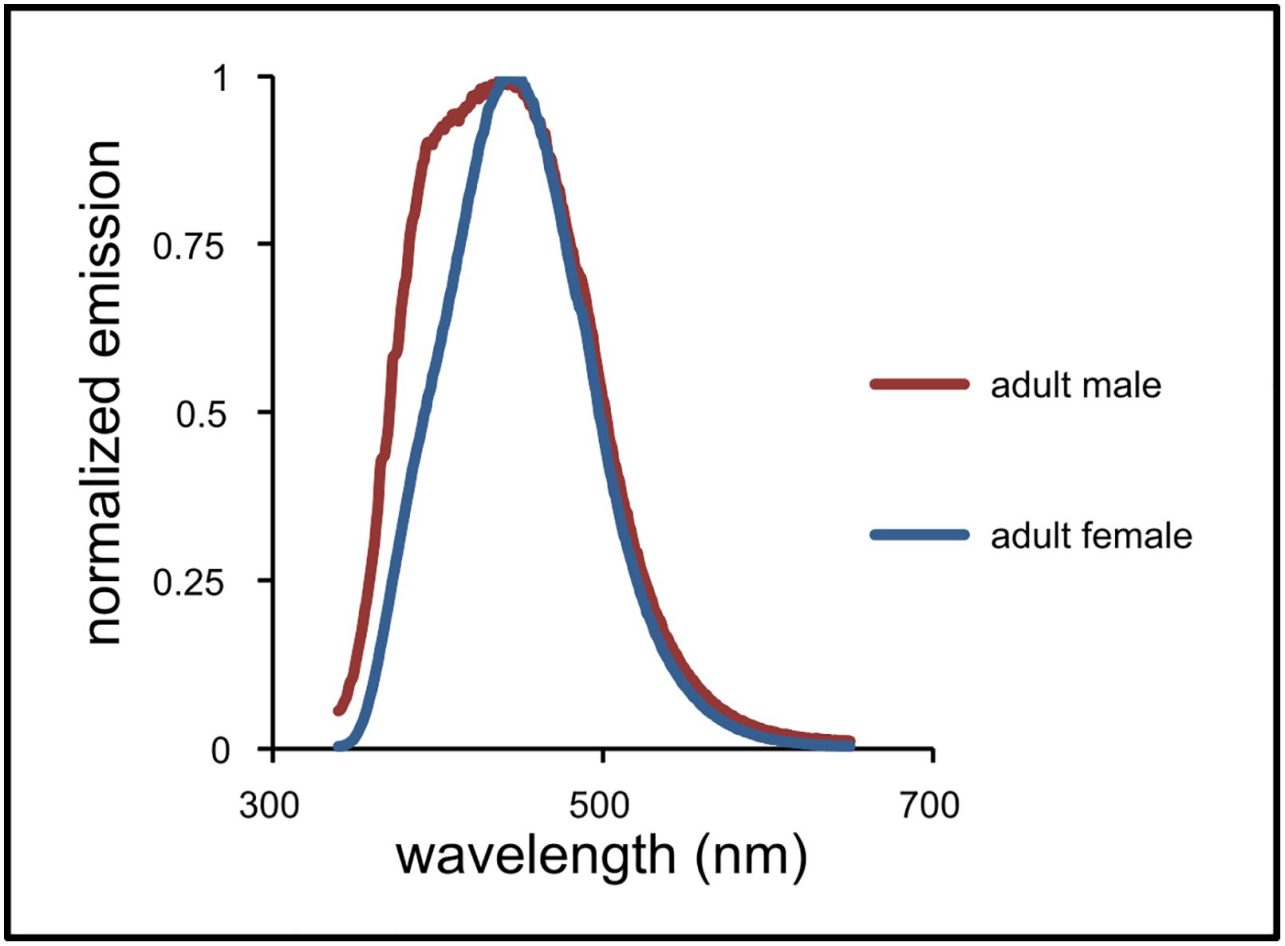
Normalized emission of fluorophores from one representative adult male and one representative adult female *M*. *vatia* excited with 330-nm light.

**Fig 5:**
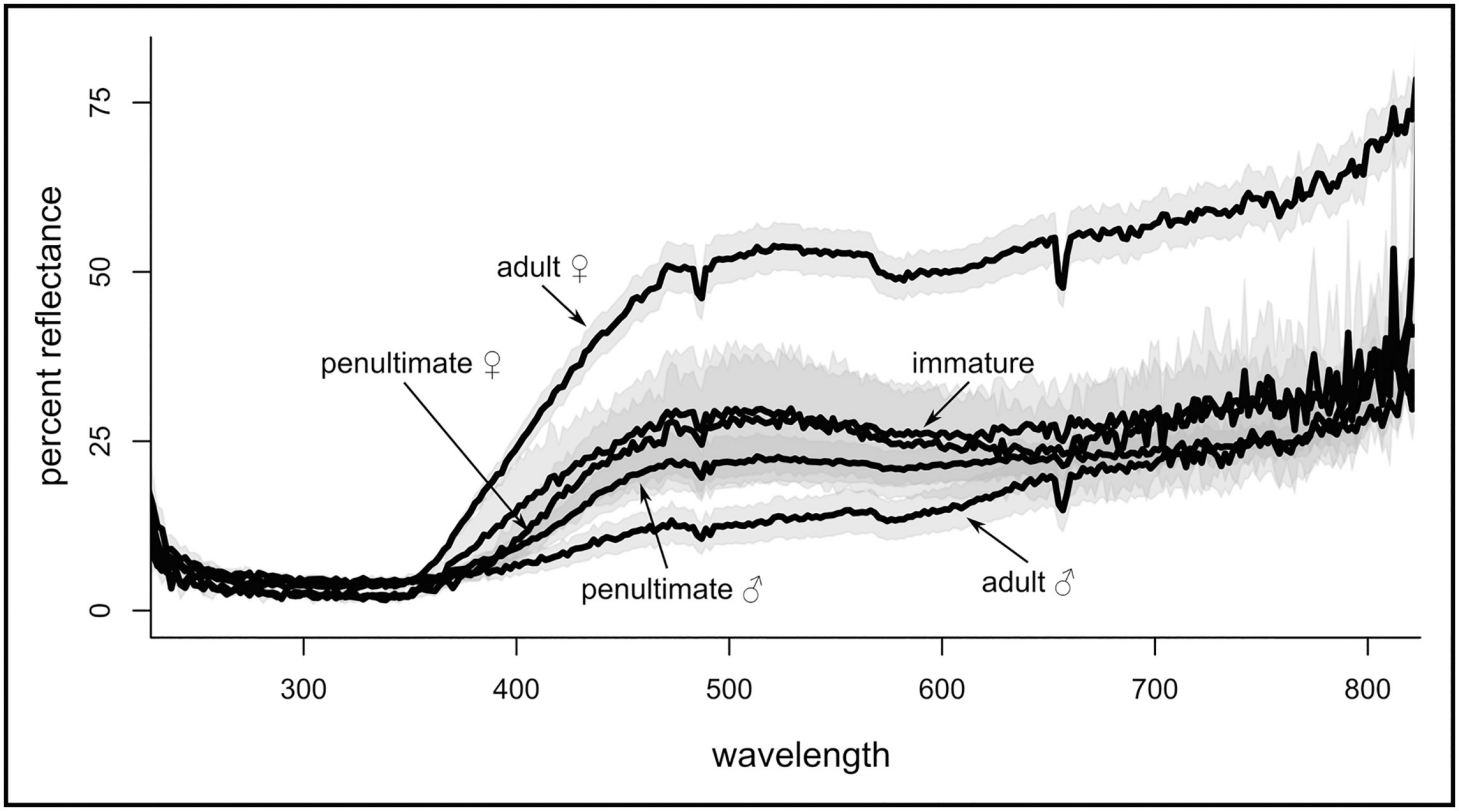
Reflectance measurements from M. *vatia* abdomens at various life stages. (Adult females n = 17; adult males n = 5; penultimate females n = 3; penultimate males n = 9; immatures n = 2). For each individual, ten measurements were averaged together from different parts of the dorsal surface of the abdomen. The data used to generate this figure is provided in S2 Dataset.

The peak emission wavelengths of the abdominal extracts are between about 350–550 nm (Fig 4), a range that encompasses the peak of reflectance seen in female and immature spiders (Fig 5). This strongly suggests that fluorophores found in the abdominal extracts are the same fluorophores as those expressing fluorescence at the surface of the spider, or that the fluorophores, if different, share the same spectral characteristics. The similarity in fluorophores, together with the differences in external fluorescence, suggest that selection has shaped fluorescence by influencing either how near the fluorophores approach the surface of the cuticle or how opaque the cuticle is to the penetration of light—and that this can and does change over the lifetime of a male spider.

### Fluorophores present in *Misumena vatia*

Fluorophores extracted from *M*. *vatia* showed peak excitation at 330 nm. When excited at 330 nm, the peak emission wavelengths of the fluorophores from the abdominal extracts are between about 350–550 nm (Fig 4). We also found that the peak excitation wavelengths of these fluorophores (330 nm) coincided closely with the excitation wavelengths that produced the brightest images (340 nm). The fluorophores of males and females showed similar emission peaks.

### Reflectance

We averaged abdominal reflectance measurements for individuals within each sex and life stage, and present them for qualitative comparison. The abdomens of adult female spiders showed the highest overall reflectance of wavelengths between 350 and 800 nm (Fig 5). This encompasses the range of the peak emission of fluorophores seen in female and male spiders (Fig 4). In contrast to females, the abdomens of adult males were on average the least reflective (Fig 5). Average reflectance of immature spiders was similar to that of penultimate females, and both were more reflective than penultimate male spiders. Additionally, there is a peak of reflectivity at about 450–550 nm seen in adult and penultimate females and immature spiders that is absent or greatly reduced in adult male spiders.

## Discussion

Our data show that the degree of externally expressed fluorescence in the crab spider *M*. *vatia* differs significantly between males and females, and that this changes over the course of a spider’s lifetime. However, the fluorophore emission profiles of males and females are very similar, implying that the fluorophores of each sex possess the same physiological properties and constraints. There are several potential explanations for these findings. The distinct sexual dimorphism in the expression of fluorescence, and the differences across life stages, may suggest that selection has shaped fluorescence expression in *M*. *vatia*. Alternatively, externally expressed fluorescence may serve no biological function and fluorophores may be a non-selective byproduct of some metabolic process unrelated to fluorescence. Below we pose several hypotheses that could explain our findings, and discuss whether or not our data are consistent with each hypothesis.

### Is fluorescence expression non-adaptive?

Our data show that fluorophores do not differ substantially between males and females. Therefore, it is the expression of fluorescence that changes across life stages as spiders mature, resulting at maturity in sexual dimorphism in fluorescence. Perhaps selection may be acting differently on males and females to up-regulate or down-regulate certain metabolic processes, and fluorescence expression is modified simply as a by-product of selection on these processes. For example, perhaps sexes differ in development of the cuticle as spiders molt to adulthood, such that in males, the cuticle prevents fluorophores from being exposed to wavelengths of light that would stimulate them to fluoresce. Differences in cuticle transmittance have been found to affect coloration among color phenotypes of female crab spiders [26], and perhaps differences in cuticle transmittance could exist between the sexes. Teasing apart non-adaptive explanations for fluorescence from adaptive explanations could prove difficult, depending on how tightly linked fluorescence expression may be to metabolic processes under selection.

### Do fluorophores offer photo-protection (A sunscreen effect)?

Because the sun emits light at high-energy ultraviolet wavelengths and some of this radiation arrives unattenuated at the Earth’s surface, organisms exposed to sunlight are at some risk of tissue damage, DNA damage, and cancer [27]. The fluorophores of *M*. *vatia* convert high-energy UV light to lower-energy visible light, so it would seem reasonable to speculate that fluorescence may serve a photo-protective function for these sun-exposed predators.

Such a “sunscreen hypothesis” has been proposed for several coral species that frequently become exposed to the sun during low tides [28, 29]. A role for fluorophores in protecting spiders against UV light has likewise been proposed [30]. It has been found that ommochromes, which protect against UV light, are the primary pigment present in *M*. *vatia* [31]. The precursors to the formation of ommochrome pigments are fluorescent, and are present in white colored *M*. *vatia* [31, 32]. If the primary role of fluorescence in *M*. *vatia* is photo-protection, we might predict that the individuals with the greatest need to avoid DNA damage (adult females carrying developing eggs) should fluoresce most brightly.

The abdomens of adult females can become extremely distended with eggs [33]. Because adults do not molt further, the cuticle must stretch to accommodate the eggs, presumably becoming thinner and more transparent to light, including dangerous UV wavelengths. It would seem, then, that adult females (and their eggs) would benefit from increased fluorescence if its function were protection from UV light. However, this is not what our data show; instead we find that adult females fluoresce slightly less brightly than penultimate females. Thus, while we cannot rule out that fluorescence protects against UV radiation in *M*. *vatia*, it seems unlikely that its sole function in this species is photo-protection.

### Does fluorescence enable crypsis?

Many spiders use visual signals to manipulate the behavior of conspecifics or heterospecifics [34]. As *M*. *vatia* does not seem to use visual sexual displays [35], our visual signaling hypotheses focus on potential interactions between *M*. *vatia* and its predators and/or prey. Specifically, we might expect these crab spiders to use fluorescence-enhanced coloration for crypsis and/or for prey attraction.

Many spiders across a diversity of families are masters of camouflage, with striking examples from the crab spider family Thomisidae [36]. Crab spiders may be highly cryptic in their surroundings, possibly to avoid being detected by prey [16, 37, 38]. Some flowers are known to be UV-reflective (e.g. [39]) and some are known to fluoresce [40, 41]. It is possible that a spider’s externally expressed fluorescence helps to match the spider to its background. However, whether or not *Misumena* are cryptic depends both on the background against which the spider is viewed and on the visual system of the animal observing it. Surveys of European populations of *M*. *vatia* and the plants on which they hunt have revealed that bees (a primary prey source) are able to discern *M*. *vatia* on most flowers at close range [42] but that these spiders remain cryptic to their bee prey at greater distances [42]. Likewise, dipterans do not readily avoid spiders on flowers [43], suggesting that the spiders may remain cryptic to them.

Crypsis may also protect spiders from their visually oriented predators. Indeed, *M*. *vatia* on flowers are thought likely to be cryptic to potential avian predators when viewed from a distance [42]. Avian predation pressure could potentially have selective consequences for any coloration that *M*. *vatia* expresses or modifies via fluorescence.

For immature *M*. *vatia* of small body sizes, jumping spiders represent a serious predation risk [44, 45]. Immature *M*. *vatia* instars would presumably benefit from effective camouflage to hide from visually keen salticid predators [46, 47].

### Does fluorescence attract prey?

Another hypothesis is that *M*. *vatia* might use external fluorescence to generate or accentuate a visual signal that prey organisms find attractive. For example, fluorescent tentacles of cnidarian hydromedusas have been found to attract the fish that these animals typically prey upon [48]. Several studies have found evidence that in some spiders body coloration and pattern serve to attract prey [49, 50], and that ultraviolet reflectance in particular serves as a prey attractant in crab spiders [51, 52]. A study of crab spiders in Australia found that honeybees were more likely to land on flowers with large spiders that were highly UV-reflective, or on flowers with small spiders that were minimally UV-reflective, than on flowers with spiders showing other trait combinations [53]. These findings are consistent with the idea that crab spider coloration may mimic the UV-reflective nectar guides that have evolved in some flowers to attract pollinators that perceive ultraviolet light. As a result, large spiders that reflect ultraviolet light—or those that emit bright UV fluorescence—may enjoy enhanced success at capturing pollinators that see in the UV and that perceive them to be floral nectar guides.

If this is the case, we would expect prey species to be preferentially drawn to flowers containing crab spiders. To date, there are no experimental data showing this with *M*. *vatia*. However, to our knowledge, field studies of *Misumena* and plant-prey interactions (such as have been performed by Dukas and Morse [54, 55] or Brechbühl et al [56]) have not yet been conducted on populations in the Pacific Northwestern USA. Closely related and morphologically similar thomisid species have been shown to be quite different spectrally, with different effects on prey species [37, 51, 57, 58]. Thus, it is possible that populations of *Misumena* in the Pacific Northwest might well possess fluorophores that emit wavelengths of light attractive to insect prey in this region.

Indeed, a study of a diverse assemblage of bees in the Pacific Northwest found that they are significantly attracted to fluorescent traps that absorb UV light and emit peak wavelengths at about 430 nm [59]. The fluorophores of the *Misumena* in our study show peak emission profiles (Fig 4) in the same range as the wavelengths preferred by the bees assayed by Rao and Ostroverkhova [59]. Moreover, some dipterans seem to be attracted to emission wavelengths similar to those of the *M*. *vatia* fluorophores [60].

### How might fluorescence function?

Our hypotheses for the function(s) of fluorescence in crab spiders are not mutually exclusive. For instance, fluorescence might help a spider hide from predators or prey while also helping it to mimic a flower’s nectar guide and thereby attract prey. Or fluorescence might serve one or more visual signaling functions while fluorophores also help protect against damage from UV radiation.

Beyond the complexity that can stem from multiple overlapping functions, it seems reasonable to expect further complexity to arise from the differences in ecology and behavior between sexes and among age classes. For example, male *M*. *vatia* are smaller than females [61], and differ in their behavior, spending most of their time searching for a mate [62]. Male spiders also fluoresce significantly less than female spiders. With no need to eat a great deal of food to produce eggs, adult males may be able to afford to pass up any advantages of bright fluorescence in order to maintain a more subdued coloration that reduces their visibility during their nomadic wanderings.

Conversely, small-bodied immature crab spiders may benefit from investing in crypsis in order to minimize the risk of predation by jumping spiders. If fluorescence enhances an immature spider’s camouflage atop flowers, this benefit may well outweigh the risk it faces of injury from large hymenopterans visiting those flowers.

Because of such complexity, future research on fluorescence in spiders will need to take into account the variation in behavior, ecology, and life history between the sexes and among developmental stages. Thus far, vision-related research with spiders and their substrates, predators, and prey has focused on adult females. Applying knowledge gained from close study of various aspects of the biology of spiders [42, 62–64]—male and female, young and old—will yield a more complete picture of what roles fluorescence may play in spider biology.

## Conclusions

The existence of externally expressed fluorescence in spiders has been puzzling since its initial discovery. Our unique instrumentation design allowed us to specifically analyze the emissions from fluorophores in intact spiders when excited with wavelengths and intensity of light equivalent to what these spiders would experience on a sunny day in the Pacific Northwestern USA. Our findings suggest that for *M*. *vatia*, a species of sun-exposed crab spider, externally expressed fluorescence contributes to the overall coloration that may be perceived by its predators and prey. Given what is known about the ecology of this spider, we suggest that the portion of *M*. *vatia’s* coloration that results from fluorescence may play a role in prey attraction, crypsis for predation avoidance, and/or crypsis for prey acquisition. Our work also highlights the importance of understanding the developmental context of visual signals, especially in organisms that utilize different ecological niches during different life stages. Finally, we suggest that comprehending signaling processes in animals involves understanding the underlying physics of the signal, as well as the ability of the receiver to perceive it. The increasingly widespread adoption of techniques that can objectively measure and quantify coloration will advance this endeavor.

## Supporting information

S1 Fig Sunlight and ultraviolet light source absolute irradiance. (A) Absolute irradiance on a sunny day in June 2011, Portland State University, Portland, Oregon. (B) Absolute irradiance of 340-nm LED used in all fluorescence photography.
(TIF)

S2 Fig Schematic of fluorescence photography instrumentation. (1) Light is generated by interchangeable UV LED and travels through optics tube. (2) Light hits precisely angled dichroic beam splitter filter, and is directed down to the specimen. (3) Fluorophores in specimen are excited by UV light and emit visible wavelengths. (4) Visible light passes through beam splitter and passes up to blocking filter. (5) Blocking filter (optional) filters out any wavelengths other than spider fluorophore emission wavelengths. (6) Remaining wavelengths pass up into microscope for focusing and to the camera for capture.
(TIF)

S1 Table Kruskal-Wallis results for average fluorescent intensities of all body parts, using a blocking filter that allowed wavelengths above 420 nm to reach the camera. An asterisk indicates a p < 0.05. Legs of immature spiders were not imaged due to small size. (Adult females n = 15; adult males n = 9; penultimate females n = 4; penultimate males n = 9; immature spiders n = 9)
(DOCX)

S2 Table Results of post-hoc Dunn’s tests for differences in fluorescence intensity of each body part, using a blocking filter (< 420 nm). Asterisks and yellow shading indicate p < 0.05. Legs of immature spiders were not imaged due to small size.
(DOCX)

S1 Dataset Average fluorescence intensity of each spider measured, for each body part. Fluorescence intensity was measured with and without blocking filters (on different tabs). Body parts are abbreviated as follows: ab = abdomen; ct = cephalothorax; rt1 = right leg one.
(XLS)

S2 Dataset Spectrometer readings for each spider measured. The wavelengths of light recorded for each individual are presented on different tabs of the dataset, according to sex and life stage.
(XLS)
