# Supplementary figures and images for: Females are the brighter sex: Differences in external fluorescence across sexes and life stages of a crab spider
Source: PLoS One. 2017 May 3;12(5):e0175667. doi: 10.1371/journal.pone.0175667 (PMC5414973; doi:10.1371/journal.pone.0175667)

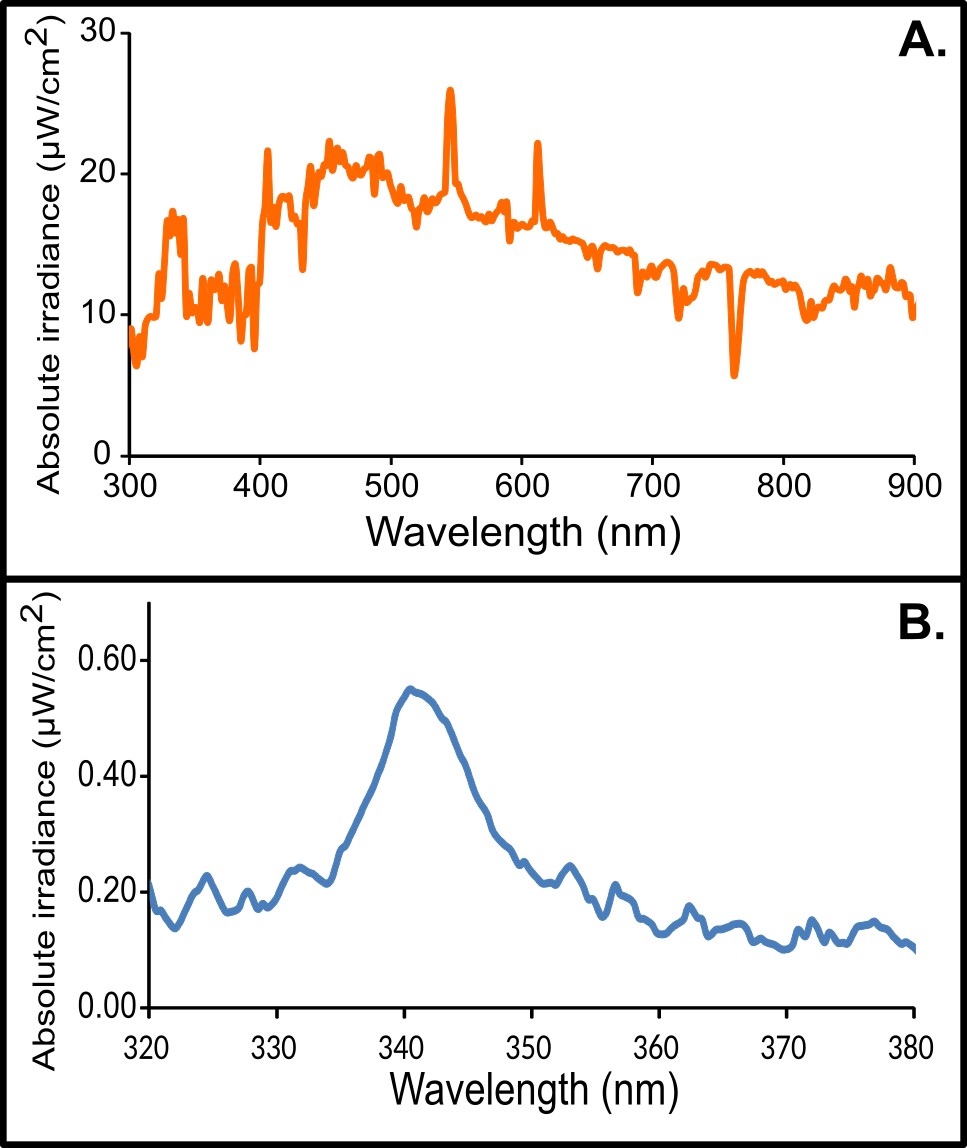

Supplement: S1 Fig — (A) Absolute irradiance on a sunny day in June 2011, Portland State University, Portland, Oregon. (B) Absolute irradiance of 340-nm LED used in all fluorescence photography. (TIF) [file pone.0175667.s001.tif]

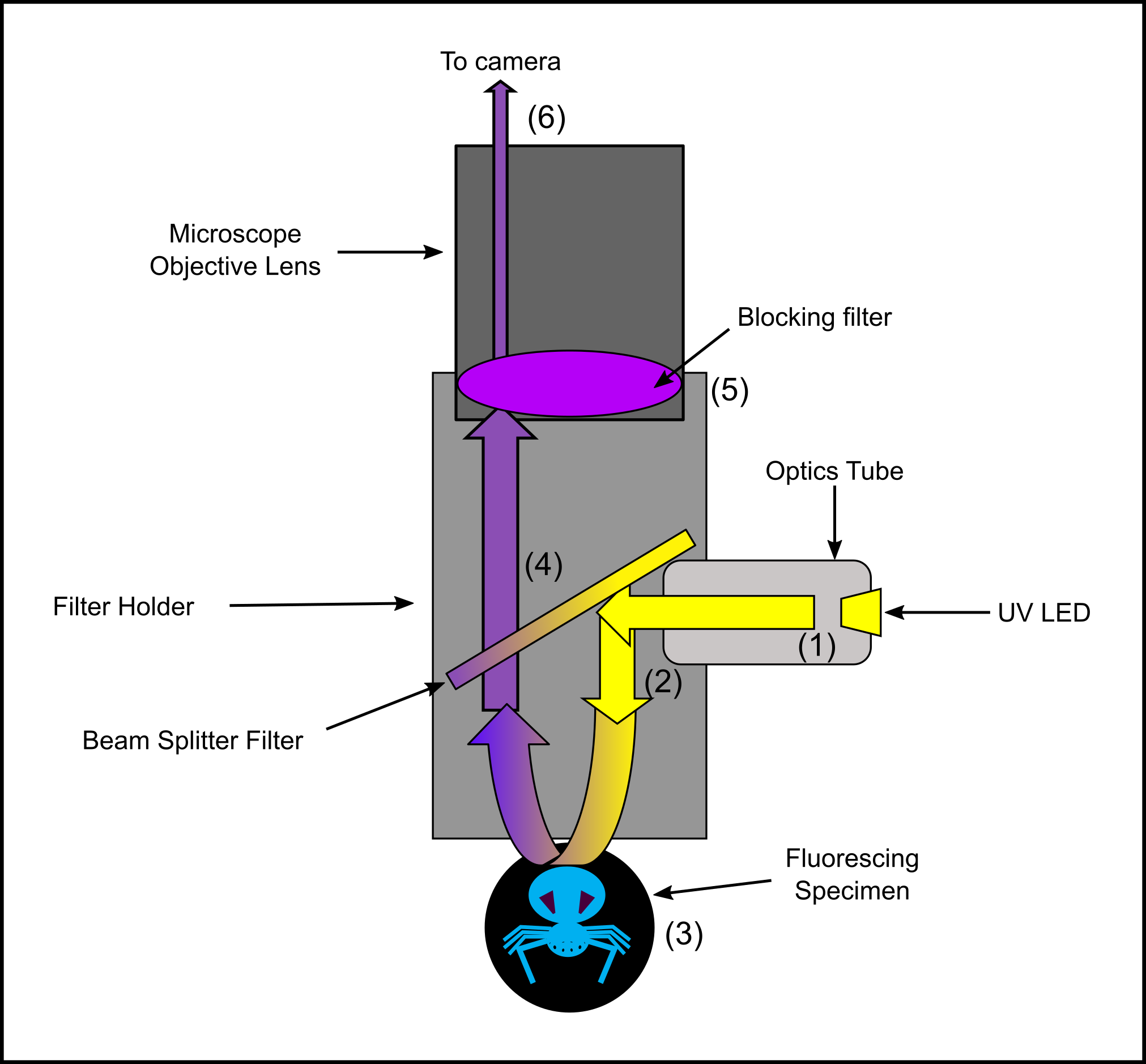

Supplement: S2 Fig — (1) Light is generated by interchangeable UV LED and travels through optics tube. (2) Light hits precisely angled dichroic beam splitter filter, and is directed down to the specimen. (3) Fluorophores in specimen are excited by UV light and emit visible wavelengths. (4) Visible light passes through beam splitter and passes up to blocking filter. (5) Blocking filter (optional) filters out any wavelengths other than spider fluorophore emission wavelengths. (6) Remaining wavelengths pass up into microscope for focusing and to the camera for capture. (TIF) [file pone.0175667.s002.tif]
