# Supplementary material for: Females are the brighter sex: Differences in external fluorescence across sexes and life stages of a crab spider
Source: PLoS One. 2017 May 3;12(5):e0175667. doi: 10.1371/journal.pone.0175667 (PMC5414973; doi:10.1371/journal.pone.0175667)
Supplement: S1 Table — An asterisk indicates a p < 0.05. Legs of immature spiders were not imaged due to small size. (Adult females n = 15; adult males n = 9; penultimate females n = 4; penultimate males n = 9; immature spiders n = 9) (DOCX) [file pone.0175667.s003.docx]

**S1 Table. Kruskal-Wallis results for average fluorescent intensities of all body parts, using a blocking filter that allowed wavelengths above 420 nm to reach the camera.** An asterisk indicates a p < 0.05. Legs of immature spiders were not imaged due to small size. (Adult females n = 15; adult males n = 9; penultimate females n = 4; penultimate males n = 9; immature spiders n = 9)

| **Average Intensity** | X^2^ | X^2^_crit_ | df | p |
| --- | --- | --- | --- | --- |
| abdomen | 18.106 | 9.48 | 4 | 0.001* |
| cephalothorax | 33.497 | 9.48 | 4 | << 0.001* |
| right leg 1 | 24.58 | 7.81 | 3 | << 0.001* |
