# Supplementary material for: Females are the brighter sex: Differences in external fluorescence across sexes and life stages of a crab spider
Source: PLoS One. 2017 May 3;12(5):e0175667. doi: 10.1371/journal.pone.0175667 (PMC5414973; doi:10.1371/journal.pone.0175667)
Supplement: S2 Table — Asterisks and yellow shading indicate p < 0.05. Legs of immature spiders were not imaged due to small size. (DOCX) [file pone.0175667.s004.docx]

**S2 Table. Results of post-hoc Dunn’s tests for differences in fluorescence intensity of each body part, using a blocking filter (< 420 nm).** Asterisks and yellow shading indicate p < 0.05. Legs of immature spiders were not imaged due to small size.

| **abdomen** | adult male | penultimate female | penultimate male | immature |
| --- | --- | --- | --- | --- |
| adult female | 0.007* | 0.895 | 1.000 | 1.000 |
| adult male | - | 0.003* | 0.017* | 0.043* |
| penultimate female |  | - | 0.907 | 0.849 |
| penultimate male |  |  | - | 1.000 |
| **cephalothorax** | adult male | penultimate female | penultimate male | immature |
| adult female | << 0.001* | 0.902 | 0.007* | 0.423 |
| adult male | - | 0.003* | 0.411 | 0.007* |
| penultimate female |  | - | 0.154 | 0.616 |
| penultimate male |  |  | - | 0.423 |
| **right leg 1** | adult male | penultimate female | penultimate male |  |
| adult female | << 0.001* | 0.617 | 0.045* |  |
| adult male | - | 0.033* | 0.092 |  |
| penultimate female |  | - | 0.617 |  |
